# Supplementary material for: Investigation of the impact of commonly used medications on the oral microbiome of individuals living without major chronic conditions
Source: PLoS One. 2021 Dec 9;16(12):e0261032. doi: 10.1371/journal.pone.0261032 (PMC8659300; doi:10.1371/journal.pone.0261032)
Supplement: S1 Fig — Alpha diversity represented by (A) Shannon diversity and (B) Evenness, and beta diversity as represented by (C) Bray-Curtis dissimilarity, and (D) unweighted UniFrac. (PDF) [file pone.0261032.s001.pdf]

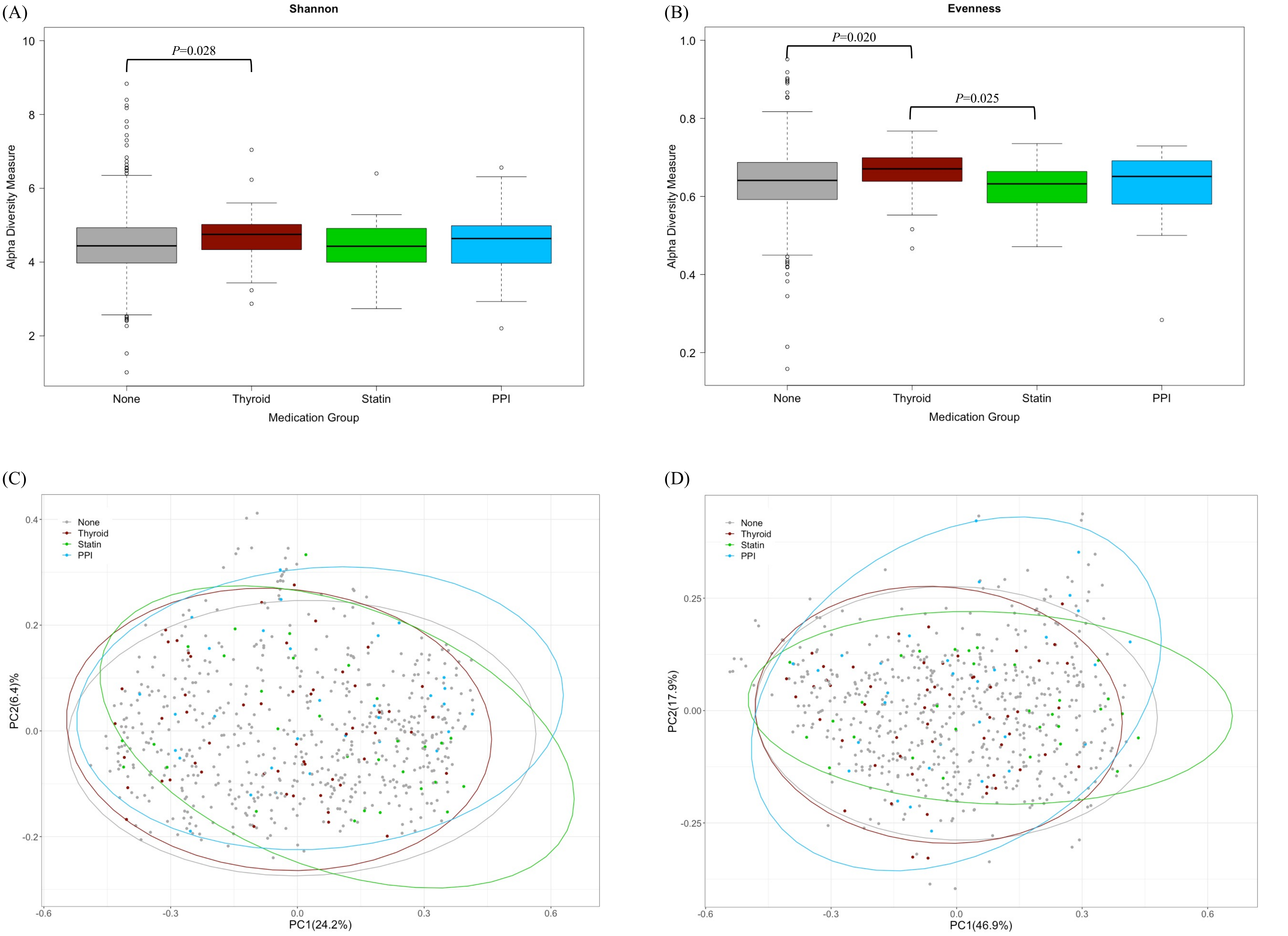

S1 Fig. Microbial diversity among participants only taking Thyroid Hormones, Statins, or PPIs compared to participants taking no medication. Alpha diversity represented by (A) Shannon diversity and (B) Evenness, and beta diversity as represented by (C) Bray-Curtis dissimilarity, and (D) unweighted UniFrac. *P*-values above box plots indicate the results of Dunn's tests with Bonferroni correction. None n=546; Thyroid n=54; Statin n=30; PPI, n=31.
