# Supplementary material for: Investigation of the impact of commonly used medications on the oral microbiome of individuals living without major chronic conditions
Source: PLoS One. 2021 Dec 9;16(12):e0261032. doi: 10.1371/journal.pone.0261032 (PMC8659300; doi:10.1371/journal.pone.0261032)
Supplement: S2 Fig — Beta-diversity represented by Principal Coordinates Analysis plots based on weighted uniFrac distances among participants taking no medication and those taking (A) thyroid, (B) statin, and (C) PPI. (PDF) [file pone.0261032.s002.pdf]

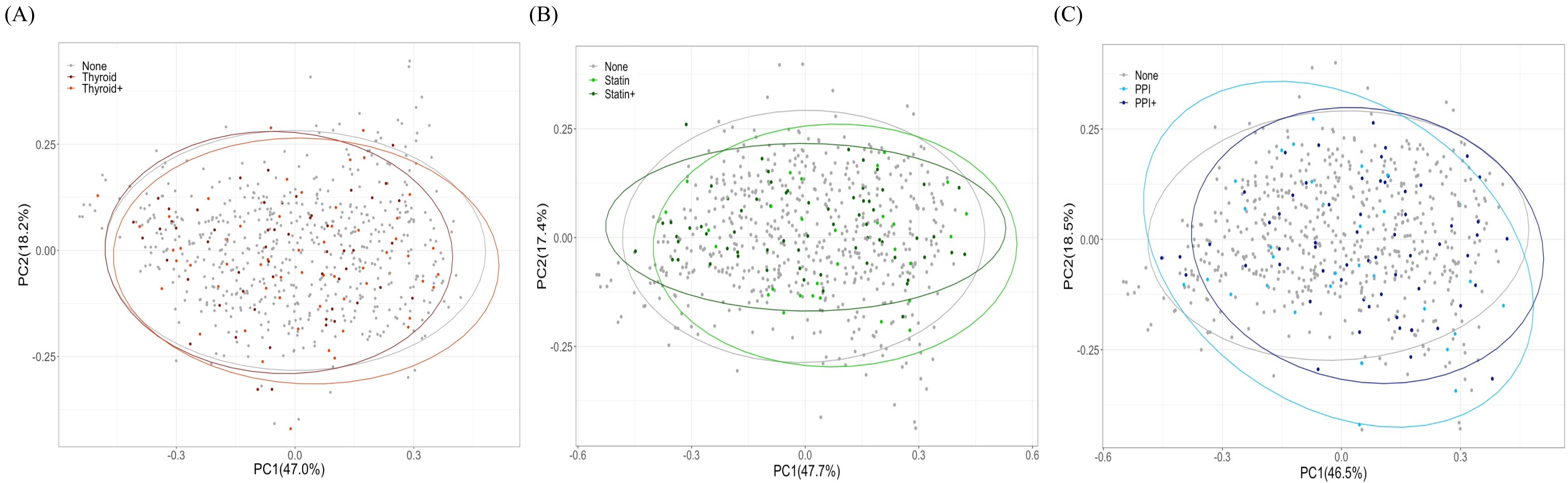

S2 Fig. Beta diversity analyses among (A) thyroid, (B) statin, and (C) PPI users and non-medication users are represented by Principal Coordinates Analysis plots based on Bray-Curtis dissimilarity. None represents participants taking no medications (n=546); Thyroid represents participants only taking Thyroid Hormone medication (n=54); Thyroid+ represents participants taking Thyroid Hormone medication plus other medication(s)(n=58); Statin represents participants only taking Statin medication(n=30); Statin+ represents participants taking Statin medication plus other medication(s) (n=65); PPI represents participants only taking PPI medication (n=31); PPI+ represents participants taking PPI medication plus other medication(s)(n=61).
