# Supplementary material for: Investigation of the impact of commonly used medications on the oral microbiome of individuals living without major chronic conditions
Source: PLoS One. 2021 Dec 9;16(12):e0261032. doi: 10.1371/journal.pone.0261032 (PMC8659300; doi:10.1371/journal.pone.0261032)
Supplement: S3 Fig — Differentially abundant genera in (A) thyroid hormone users, and (B) statin users compared to participants taking no medication, controlling for covariates (sex, age, and BMI). (PDF) [file pone.0261032.s003.pdf]

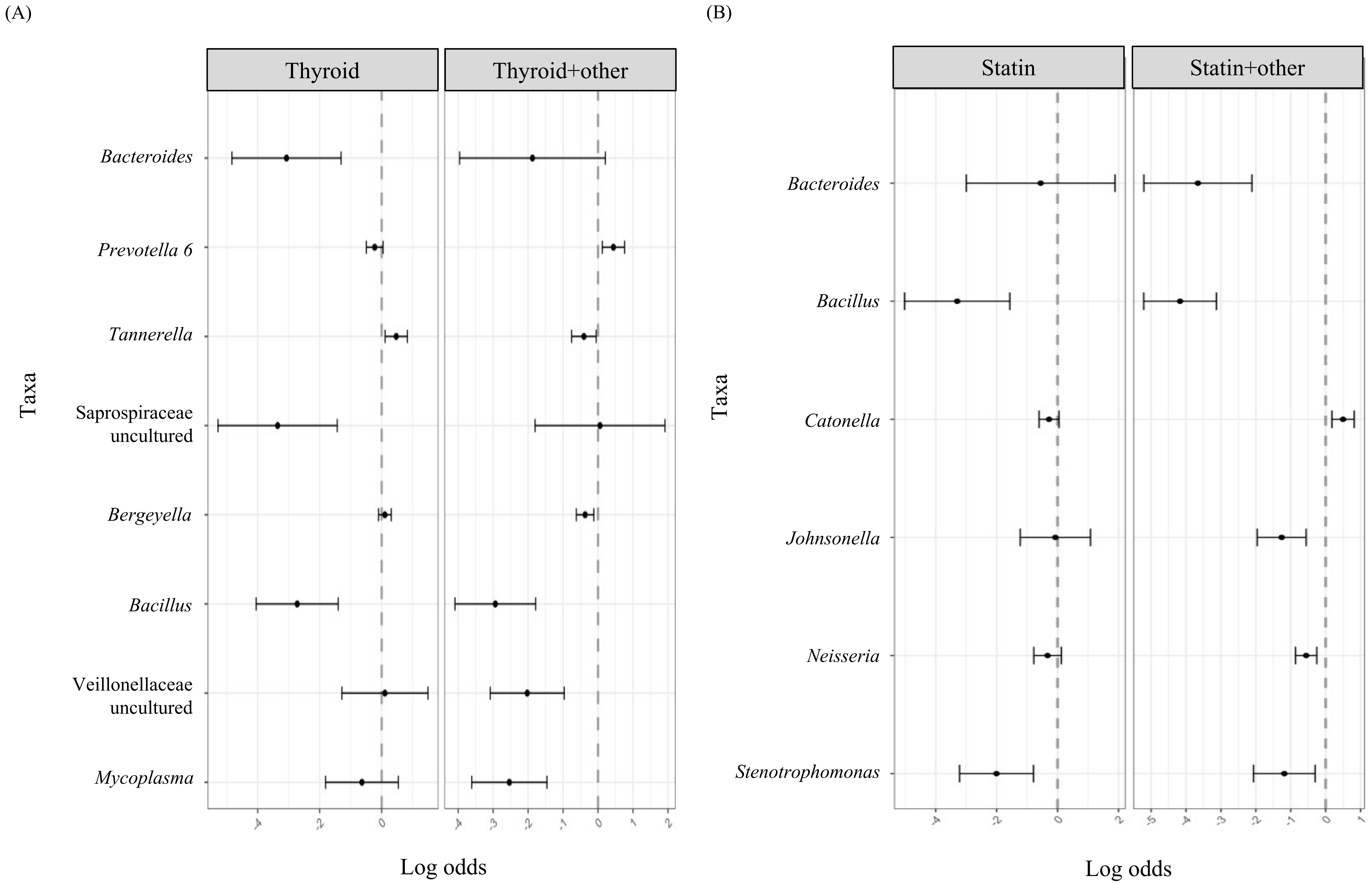

S3 Fig. Differentially abundant genera in (A) thyroid hormone users, and (B) statin users compared to participants taking no medication, controlling for covariates (sex, age, and BMI). Results were adjusted by False Discovery Rate (FDR) using Benjamini and Hochberg method and genera meeting an FDR of q=0.1 are presented. No medication (n=546); Thyroid represents participants only taking Thyroid Hormone medication (n=54); Thyroid+ represents participants taking Thyroid Hormone medication plus other medication(s) (n=58); Statin represents participants only taking Statin medication (n=30); Statin+ represents participants taking Statin medication plus other medication(s) (n=65).
