# Supplementary material for: Investigation of the impact of commonly used medications on the oral microbiome of individuals living without major chronic conditions
Source: PLoS One. 2021 Dec 9;16(12):e0261032. doi: 10.1371/journal.pone.0261032 (PMC8659300; doi:10.1371/journal.pone.0261032)
Supplement: S1 Table — (PDF) [file pone.0261032.s004.pdf]

S1 Table. Characteristics of participants taking none, one, or multi medications

Group	Sex -male/female<br>Count(%)	Age -years<br>Median (IQR)	BMI - kg/m^2<br>Median (IQR)
None	211(33)/433(67)	56 (49-62)	27 (24-30)
Single	90(28)/228(72)	56 (50-61)	27 (24-30)
Multi	67(27)/185(73)	58 (52-62)*⁺	27 (25-30)*

*Significantly different from None (P<0.05).

⁺Significantly different from Single (P<0.05).
