# Supplementary material for: Investigation of the impact of commonly used medications on the oral microbiome of individuals living without major chronic conditions
Source: PLoS One. 2021 Dec 9;16(12):e0261032. doi: 10.1371/journal.pone.0261032 (PMC8659300; doi:10.1371/journal.pone.0261032)
Supplement: S2 Table — (PDF) [file pone.0261032.s005.pdf]

S2 Table. Characteristics of participants taking the most frequently reported medications alone or in combination

Group	Sex -male/female Count(%)	Age -years Median (IQR)	BMI - kg/m^2 Median (IQR)
None	211(33)/433(67)	56 (49-62)	27 (24-30)
Thyroid hormone only	5(8)/55(92)*	55 (50-59)	27 (23-30)
Thyroid hormone plus	6(9)/61(91)*	59 (54-62)*	27 (23-30)
Statin only	24(67)/12(33)*	59 (56-63)*	28 (25-29)
Statin plus	30(40)/45(60)$^+$	61 (57-64)*	28 (26-30)*
Proton Pump inhibitor (PPI) only	11(29)/27(71)	54 (49-61)	29 (26-32)*
PPI plus	19(28)/49(72)	58 (52-62)	28 (26-30)*

*Significantly different from None ($P<0.05$).

$^+$Significantly different from Statin only ($P<0.05$).
