# Supplementary material for: Investigation of the impact of commonly used medications on the oral microbiome of individuals living without major chronic conditions
Source: PLoS One. 2021 Dec 9;16(12):e0261032. doi: 10.1371/journal.pone.0261032 (PMC8659300; doi:10.1371/journal.pone.0261032)
Supplement: S3 Table — (PDF) [file pone.0261032.s006.pdf]

**S3 Table.** Differentially abundant genera in saliva of single and multi-medication users

| Genera | Single Medication Users | | Multi Medication Users | |
|---|---|---|---|---|
| | Log Odds coefficient (SE) | *p*-value | Log Odds coefficient (SE) | *p*-value |
| Saprospiraceae uncultured | 1.610 (0.639) | 0.012 | 2.273 (0.690) | 0.001 |
| *Bacillus* | -2.042 (0.374) | <0.001 | -1.104 (0.389) | 0.005 |
| *Johnsonella* | -0.170 (0.201) | 0.399 | -0.902 (0.241) | <0.001* |
| *Actinobacillus* | -0.060 (0.329) | 0.855 | -1.286 (0.375) | <0.001* |
| *Stenotrophomonas* | -0.084 (0.352) | 0.811 | -1.046 (0.339) | 0.002 |
| *Mycoplasma* | -1.802 (0.345) | <0.001 | -2.394 (0.340) | <0.001 |

Above values represent abundance coefficients of single and multi-medication users compared to non-medication users by Corncob analysis.

*Statistically different from non-medication users by both Corncob and MaAsLin2 analysis (q-value < 0.05).

There was no overlap between the above genera and additional differential abundance test using ALDEx2, or ANCOM2.
