# Supplementary material for: Investigation of the impact of commonly used medications on the oral microbiome of individuals living without major chronic conditions
Source: PLoS One. 2021 Dec 9;16(12):e0261032. doi: 10.1371/journal.pone.0261032 (PMC8659300; doi:10.1371/journal.pone.0261032)
Supplement: S4 Table — (PDF) [file pone.0261032.s007.pdf]

S4 Table. Differentially abundant genera in saliva of Thyroid medication users

Genera	Thyroid		Thyroid+	
	Log Odds coefficient (SE)	*p*-value	Log Odds coefficient (SE)	*p*-value
Bacteroides	-3.069 (0.898)	<0.001	-1.874 (1.063)	0.078
Prevotella 6	-0.224 (0.138)	0.105	0.441 (0.162)	0.007
Tannerella	0.471 (0.184)	0.011	-0.404 (0.179)	0.024
Saprospiraceae uncultured	-3.356 (0.981)	<0.001	0.057 (0.948)	0.952
Bergeyella	0.104 (0.105)	0.319	-0.370 (0.128)	0.004
Bacillus	-2.725 (0.675)	<0.001	-2.937 (0.589)	<0.001
Veillonellaceae uncultured	0.106 (0.708)	0.882	-2.024 (0.540)	<0.001
Mycoplasma	-0.636 (0.599)	0.289	-2.536 (0.549)	<0.001

Above values represent abundance coefficients of Thyroid and Thyroid+ medication users compared to non-medication users by Corncob analysis.

Thyroid represents participants only taking Thyroid Hormone medication; Thyroid+ represents participants taking Thyroid Hormone medication plus other medication(s).

There was no overlap between the above genera and additional differential abundance test using by MaAsLin2, ALDEx2, or ANCOM2.
