# Supplementary material for: Investigation of the impact of commonly used medications on the oral microbiome of individuals living without major chronic conditions
Source: PLoS One. 2021 Dec 9;16(12):e0261032. doi: 10.1371/journal.pone.0261032 (PMC8659300; doi:10.1371/journal.pone.0261032)
Supplement: S5 Table — (PDF) [file pone.0261032.s008.pdf]

S5 Table. Differentially abundant genera in saliva of Statin medication users

Genera	Statin		Statin+	
	Log Odds coefficient (SE)	*p*-value	Log Odds coefficient (SE)	*p*-value
Bacteroides	-0.556 (1.245)	0.655	-3.660 (0.790)	<0.001
Bacillus	-3.294 (0.881)	<0.001	-4.171 (0.531)	<0.001
Catonella	-0.281 (0.167)	0.092	0.499 (0.163)	0.002
Johnsonella	-0.073 (0.588)	0.901	-1.259 (0.358)	<0.001
Neisseria	-0.328 (0.231)	0.155	-0.558 (0.156)	0.001*
Stenotrophomonas	-2.004 (0.619)	<0.001	-1.184 (0.451)	0.009

Above values represent abundance coefficients of Statin and Statin+ medication users compared to non-medication users by Corncob analysis.

Statin represents participants only taking Statin medication; Statin+ represents participants taking Statin medication plus other medication(s).

*Trend in differential abundance from non-medication users by masslin2 (*P*=0.057).

There was no overlap between the above genera and additional differential abundance test using by MaAsLin2, ALDEx2, or ANCOM2.
